# A survey-based study of Zika virus communication preferences among pregnant women in Georgia, United States

**DOI:** 10.1186/s12884-017-1516-0

**Published:** 2017-09-26

**Authors:** Mallory K. Ellingson, Catherine M. Bonk, Allison T. Chamberlain

**Affiliations:** 1 Department of Epidemiology, Rollins School of Public Health, Emory University, 1518 Clifton Rd. NE, Atlanta, GA 30322 USA; 2 Atlanta Gynecology & Obstetrics, Decatur, USA

**Keywords:** Zika, Public health emergency, Communication, Obstetric care, Prenatal care, Social media

## Abstract

**Background:**

Because of the particularly severe perinatal outcomes associated with antenatal Zika virus infection, it is important for prenatal care providers to communicate Zika virus risks and strategies for prevention to their patients. Although face-to-face communication is ideal, clinic visits may not allow for in-depth discussion of all concerns. While previous studies have shown prenatal providers to be pregnant women’s most trusted sources of health information, there is little knowledge on what secondary communication modalities pregnant women prefer for receiving information from their providers about an evolving public health emergency.

**Methods:**

A cross-sectional, descriptive anonymous 27-item survey was distributed to pregnant women at four clinics around Atlanta, Georgia from May 5th to June 20th, 2016. The survey assessed women’s interest in and communication preferences about prenatal topics, including Zika virus. Descriptive statistics were calculated and chi-square tests were used to evaluate associations between the primary outcomes and patient characteristics.

**Results:**

Four-hundred and eight women completed the survey. The most popular resource for obtaining Zika virus information was the Centers for Disease Control and Prevention (CDC) website (73.0%). While their prenatal provider’s own website for Zika information ranked 5th among sources currently accessed for Zika information, it ranked third behind educational brochures and emails for ways in which women wanted to receive information. The characteristics of Zika virus information deemed most important were: evidence-based (87.5%), endorsed by the CDC (74.1%), and endorsed by their own provider (67.9%).

**Conclusion:**

In any public health emergency affecting pregnant women, women are going to seek advice from their obstetric providers. Because providers may lack sufficient time to discuss concerns with every patient, they may consider providing patient education in other ways. For the women included in this study, educational brochures, emails and providers’ own practice websites were preferred. Providers should consider taking greater advantage of these modalities to supplement in-person exchanges, particularly during a public health emergency.

**Electronic supplementary material:**

The online version of this article (10.1186/s12884-017-1516-0) contains supplementary material, which is available to authorized users.

## Background

Zika virus was first reported in South America in May 2015. Since then, it has spread through the Americas and the world. There is active Zika virus transmission in every country in South and Central America. The first local transmission in the United States was confirmed in July 2016 in Miami, Florida, and in November 2016, transmission of Zika virus was also reported in Brownsville, Texas [1]. Zika virus is closely related to other flaviviruses like Dengue virus and is primarily spread by mosquitoes of the genus *Aedes*, which is common across the southeast United States, particularly during the summer months of July to September [2]. However, epidemiologic data accrued during this outbreak revealed that Zika virus can also be transmitted sexually and during pregnancy from a mother to her fetus [3]. In regards to clinical presentation and outcomes Four out of five individuals infected with Zika virus are asymptomatic, and while infection typically results in mild clinical symptoms (fever, rash and joint pain), much more serious outcomes have been reported in infants born to mothers infected with the virus during pregnancy. Zika virus has been linked to severe birth defects including microcephaly and decreased brain tissue [4–7]. Because of these severe adverse outcomes, pregnant women and those considering becoming pregnant are the primary target population for education about Zika virus disease prevention and control [8].

Due to the risk of Congenital Zika Syndrome, public health officials in the U.S. have been instructing obstetric care providers to communicate Zika virus risks to their patients since January 2016 [9–12]. Many of the guidelines and recommendations issued by the Centers for Disease Control and Prevention (CDC) have been endorsed and promulgated by the American College of Obstetricians and Gynecologists (ACOG), again with a strong focus on communicating risks to pregnant women [13]. However, despite the various recommendations and travel advisories, one survey conducted in early summer 2016 found that as many as one third of pregnant women who traveled to areas with active Zika transmission were unaware of travel advisories and almost half did not know there was Zika virus transmission in the region where they traveled [14]. It was evident that Zika virus disease presented a new health communication challenge for prenatal care providers, and more research is needed on how to best discuss these types of threats with pregnant women moving forward.

Despite provider-to-patient communication being such an important aspect of risk prevention, relatively little is known on exactly how providers should communicate this information to their patients. While face-to-face conversations are ideal, ample clinic time with every patient is frequently cited as a limitation to adequate communication and discussion of all risks [15–20]. Since clinic time is limited, knowing what other modes of communication women would like their prenatal care providers to use to relay Zika virus information may be helpful for managing patient queries and more effectively disseminating public health guidance. To assist providers in conveying Zika virus-related information to their patients, this study sought to ascertain how pregnant women want to receive information about Zika virus disease﻿ from their prenatal care providers, aside from verbal communications.

## Methods

This study was granted exempt status by the Emory University Institutional Review Board. Four obstetric care practices from the Greater Atlanta Area were contacted and asked to administer the printed, anonymous survey, made available in English and Spanish. Each practice was given 100 paper copies of the survey; for the two practices with two office locations, 100 copies were delivered to each location. The survey packets consisted of two pages: a colored cover page that included the informed consent and a black-and-white copy of the survey. Front desk staff were instructed to offer the survey packets to all obstetric patients for up to 4 weeks or until 100 surveys were distributed. No information was collected on patients that declined to take the survey. The survey consisted of 27 items assessing general demographics (age range, highest education level and race/ethnicity) and interest in and preferences for receiving information from their provider about Zika virus as well as two other prenatal care topics: vaccines and safe medications. Race and ethnicity were combined into one survey question. Survey items about communication preferences provided women with six close-ended options as well as an open-ended ‘Other’ option. The communication options were selected based on previous literature about information-seeking habits of pregnant women [15, 16, 21]. All open-ended responses were analyzed for consistent themes warranting creation of any additional discrete preference categories. Information was also collected on women’s awareness of any websites and social media accounts (Facebook or Twitter) sponsored by their prenatal care practice, as well as the importance of various qualities of the educational content (endorsed by the CDC, evidence-based, endorsed by their prenatal care provider, endorsed by other mothers, or brief/succinct) provided to them by their prenatal care providers (Additional file 1). The survey was administered during the 2016 Zika epidemic; survey administration commenced at the first practice on May 5th, 2016 and concluded at the last practice on June 20th, 2016.

All data analyses were conducted using SAS version 9.3 (Cary, NC). The primary outcomes were women’s preferences for receiving information about Zika virus from their prenatal care provider. Other outcomes of interest included current sources for seeking information on Zika virus, maternal vaccination, and safe medications and degree of interest in these topics, as measured using a five-point Likert scale (not interested, somewhat interested, neutral, interested and very interested). For analyses, the 5-point Likert scale for interest was condensed to a dichotomous variable with ‘not interested,’ ‘somewhat interested’ and ‘neutral’ counting as ‘not interested’ and ‘interested’ and ‘very interested’ counting as ‘interested.’ Descriptive statistics were computed for primary analysis. The primary outcomes were also analyzed by race/ethnicity, age, education, primaparity, type of provider (ob-gyn vs. midwife) and trimester. Chi-square tests and Fisher’s exact tests were used to determine statistical significance. Significance was evaluated at α = 0.05. Crude odds ratios were calculated using unadjusted logistic regression. Adjusted odds ratios were calculated to evaluate confounding when appropriate.

## Results

In total, 408 surveys were completed. All completed surveys were in English. The largest age group represented was between 30 and 34 years of age (38.9%) and 69.8% had at least a bachelor’s degree or higher (Table 1). Most respondents were either Caucasian (40.4%) or African American/Black (37.0%). About half of the respondents were in their third trimester (50.5%) and were not pregnant for the first time (54.9%). Thirty-four percent of respondents indicated that they considered their prenatal care provider to be their primary care provider. Most respondents saw obstetricians (79.6%) compared to certified nurse midwives (11.5%); the majority (82.1%) reported seeing female providers.

**Table 1:** Patient and provider characteristics of pregnant women surveyed (*n* = 408)

Patient Characteristics	Total	
	*N*	%
Age		
18–29	132	32.3
30–34	159	38.9
35+	112	27.4
Missing	5	1.2
Education		
High School Degree or less	70	17.4
Some college	48	11.9
Bachelor Degree	117	28.6
Graduate Degree	168	41.2
Missing	5	1.2
Race		
African American/Black	151	37.0
Hispano/Latino/Chicano	20	4.9
Caucasian/White	165	40.4
Asian	48	11.8
Other	17	4.2
Missing	7	1.7
First pregnancy		
Yes	177	43.4
No	224	54.9
Missing	7	1.7
Trimester		
First	53	13.0
Second	140	34.3
Third	206	50.5
Missing	9	2.2
Type of primary prenatal care provider		
Ob-Gyn	325	79.6
Midwife	47	11.5
Both	13	3.2
Don’t Know	18	4.4
Missing	5	1.2
Sex of primary prenatal care provider		
Female	335	82.1
Male	46	11.3
Both	11	2.7
Don’t know	1	0.3
Missing	15	3.7
Considers prenatal care provider their primary provider^a^		
Yes	138	33.8
No	265	65.0
Missing	7	1.7

^a^Women were asked whether or not they consider their prenatal care provider (ob-gyn or midwife) their primary care provider (main source of health care)

All four participating practices host practice-sponsored websites and three of the four practices host a Facebook page. Only one practice has a Twitter account. About two thirds of respondents were aware that their provider has a practice website (62.8%), compared to only 9.0% of respondents who were aware of whether or not their practice sponsors a Facebook page.

Regarding Zika virus information, interest in and awareness of Zika virus was high. Nearly all women had heard of Zika virus (94.8%) and 63.0% indicated that they were interested or very interested in information about Zika virus. Pregnant women above the age of 30 were significantly more interested in Zika virus information compared to women younger than 30 years old (Age 30–34: OR = 1.99, 95% CI =1.23–3.20; Age 35+: OR = 2.95, 95% CI =1.71–5.09). Despite this high level of interest, only 40.8% of women recalled having discussed Zika virus with their providers. Compared to African-American women, Hispanic women and white women were significantly more likely to have discussed Zika virus with their providers (Hispanic: OR = 3.81, 95% CI =1.41–10.32; White: OR = 2.14 95% CI =1.34–3.42) (Table 2). Discussion with providers did not differ by trimester. Although age distribution and race/ethnicity varied between the four participating practices, adjusting for practice in the analyses did not alter the relationship between age or race and the outcomes of interest (data not shown).

**Table 2:** Provider discussion of Zika virus by race/ethnicity of pregnant women surveyed

Race/ Ethnicity	Already discussed Zika virus with provider				
	n	%	OR^a^	95% CI	*p*-value^b^
African American/Black	45	31.3	1.00	REF	REF
Hispano/Latino/Chicano	12	63.2	3.81	(1.41–10.32)	0.0085
Caucasian/White	79	46.7	2.14	(1.34–3.42)	0.001
Asian	15	31.3	1.15	(0.56–2.35)	0.70
Other	8	47.1	1.98	(0.71–5.45)	0.19

^a^Odds ratios calculated using unadjusted logistic regression

^b^Wald chi-square tests were applied to determine statistical significance

**Fig. 1:**
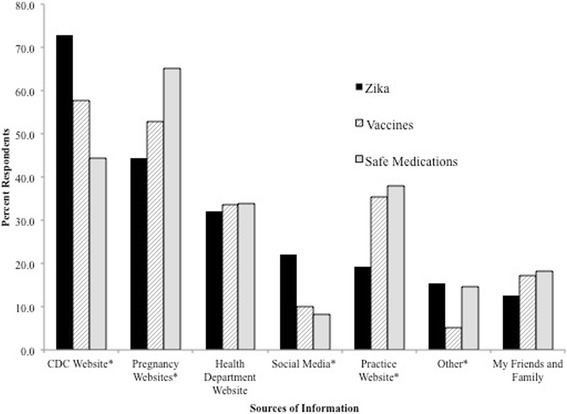
Sources used by pregnant women surveyed for obtaining information from their prenatal care provider﻿ on selected prenatal care topics. *Proportion of respondents statistically significantly differed between the three prenatal healthcare topics using a chi-square test

**Fig. 2:**
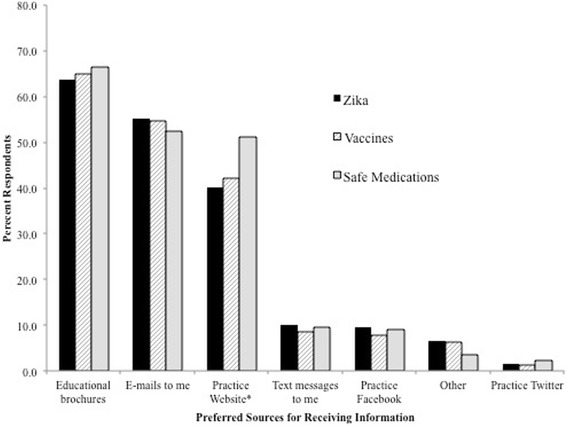
Preferred ways of receiving information about selected prenatal care topics among pregnant women surveyed. *Proportion of respondents statistically significantly differed between the three prenatal healthcare topics using a chi-square test

Aside from conversations with their prenatal providers, the top resources that women reported currently using to obtain Zika virus information were the CDC website (73.0%), other pregnancy-related websites (e.g. BabyCenter, WhatToExpect) (44.5%) and the state health department website (32.3%) (Fig. 1). A small proportion of women (15.5%) wrote in other options for their most currently used sources of information about Zika virus. The most common other responses were Google and “the news.” When asked how they would like to receive information about Zika virus from their prenatal providers, women were most interested in educational brochures (63.8%), e-mails (55.2%) and a section on their provider’s website (40.2%) (Fig. 2). Women with at least a Bachelor’s degree were significantly more interested in receiving Zika virus information through e-mails and on their provider’s practice website than women without a Bachelor’s degree (E-Mails: Bachelor’s degree, OR = 2.47, 95% CI = 1.46–4.18; Graduate degree, OR = 2.51, 95% CI = 1.54–4.08; Practice website: Bachelor’s degree, OR = 1.82, 95% CI =1.06–3.13; Graduate degree, OR = 2.11, 95% CI = 1.28–3.49). In contrast, there was little desire in being able to obtain Zika virus information via a practice-sponsored Facebook page (9.6%) or Twitter feed (1.5%). In regards to the most important qualities of the pregnancy-related information they obtain, being evidence-based (87.5%), endorsed by the CDC (74.1%), and endorsed by their own provider (67.9%) were the top three characteristics. A significantly greater proportion of women use the CDC website for information on Zika virus than for maternal vaccines and safe medications (Zika Virus: 73.0%; maternal vaccines: 57.7%; safe medications: 44.3% *p* < 0.0001). Additionally, more women look on their provider’s practice website for information about maternal vaccines and safe medications than for information on Zika virus (safe medications: 38.1%; maternal vaccines: 35.4%; Zika virus: 19.2%; *p* < 0.0001).

## Discussion

During an evolving public health threat that disproportionally affects pregnant women, it is important for prenatal care providers to know how best to communicate with their patients. We already know from research on topics like routine maternal vaccinations and general pregnancy-related information that women trust their obstetric care providers and prefer face-to-face discussions with them [15, 17, 19, 22–25]. But in situations where there is not enough time to thoroughly discuss all pertinent information and answer every question a patient has, there is a dearth of research on what secondary communication modalities women prefer and the qualities of public health information they value most. It is particularly important during an emergency situation to identify those modalities and preferences so that providers can capitalize on them to quickly and efficiently relay information to their most at-risk patient groups.

Although nearly all women in this study had heard of Zika virus, only 40% recalled discussing Zika virus with their prenatal care providers. This proportion aligns with results found in other studies conducted around the same time. From a national survey conducted between April and July 2016 among 492 women, Guo et al. found that while 97.8% of women were aware of Zika virus, only about one-third reported that their providers had discussed Zika virus with them [26]. A nationally representative poll conducted by the Kaiser Family Health Foundation in June 2016 found that 85% of Americans were aware of Zika virus. Of that 85%, 74% agreed that Zika virus presented a major risk to pregnant women but only 20% thought that Zika virus presented any threat to them or their family [27]. Another survey conducted in southeast Texas among 639 women recruited from five reproductive health clinics found that even though most women knew about Zika virus, only half were aware of the CDC travel guidelines or methods of disease transmission other than mosquito bites [28]. With such substantial gaps between mere awareness of the virus and a deeper understanding of disease risks and prevention strategies, findings from all of these studies reveal educational gaps that could have been filled.

Aside from getting information about Zika virus through conversations with their prenatal providers, we found that women are turning primarily to the Internet. They are accessing the CDC website or other pregnancy-related websites, a behavior which aligns with previous studies reporting Internet usage among pregnant women. [16, 18, 19, 21, 22, 29–32]. Furthermore, the qualities of Zika virus-related content that women valued most mirrored their information-seeking behavior; evidence-based information followed by endorsement by the CDC. The predominant use of verified, evidence-based sources like the CDC website is encouraging, however, that alone may not satisfy women’s information needs. [28] In our study, women also rated endorsement by their own provider as a very important characteristic of educational content. This desire to have public health messages validated by their personal provider makes intuitive sense and is congruent with the numerous studies that report the value women place on their own provider’s advice [22–25, 28, 33, 34].

Providers should consider all of these factors when determining what secondary communication modalities to use. For example, in this study, we found that only 19.2% of women are currently using their provider’s practice website as a source for information on Zika virus, yet over 40% indicated a desire to be able to find Zika virus information there. Additionally, significantly greater proportions of women (38.1% and 35.4%) reported already going to their providers’ websites for information on safe medications and maternal vaccines, respectively. Despite women’s interest in finding health information on their provider’s practice website and specifically their interest in finding Zika virus information there, information on Zika virus is not available on obstetric practice websites. A national review of over 900 obstetric practice websites conducted in January 2016 found that only 25% of obstetric care websites had any information about Zika virus on their websites, with only an additional 10% posting information when the review was conducted again in August 2016 following the first reports of localized transmission in the United States [35]. This lack of information about Zika virus on provider-sponsored, patient-focused websites may inadvertently leave women with the perception that Zika virus was not of the utmost importance. Since more than 85% of prenatal care providers in the US are affiliated with practices that have websites, posting information on practice websites would help fill gaps in information provision in a way that takes advantage of an existing platform that is a direct extension of the provider’s own reach [36].

Other communication modalities can also be used to convey evidence-based, verified information to pregnant women. Preferred even more than their provider’s website were brochures and emails. The CDC and other public health organizations (e.g. state health departments) have produced and continue to produce useful provider and patient-focused resources for download and circulation. What providers should consider doing when they use these resources developed by public health is to explicitly assure patients that they have reviewed and endorse the information themselves. This capitalizes on their patients’ preference for information that is endorsed by their prenatal care provider as well as evidence-based.

As providers consider utilizing secondary communication modalities, it is also important to note, “one-size may not fit all.” Certain women may prefer specific modalities over others, as evidenced in this study. Women with higher levels of education were significantly more likely to desire Zika virus information on their provider’s website or through e-mails than less well educated women. While providers may consider polling their own patient populations to determine the best ways to relay information to them, during the outset of a public health emergency, it may be just as effective and ultimately more beneficial to provide information on all existing communication outlets including the website, patient portal, social media accounts and phone systems.

In addition to having been based on responses from a highly educated patient population, this study has some other important limitations. Our sample was older, with a substantial proportion of respondents over the age of 30. Respondents were also almost exclusively white and African-American; only 4.9% of those surveyed were Hispanic. The Kaiser Family Foundation Health Tracking poll found that a greater proportion of Hispanic women were concerned about Zika virus than African-American or White women (Hispanic: 52%, African-American: 36%, White: 10%) [27]. We did not find that Hispanic women were significantly more interested in information about Zika virus, but we did find that Hispanic women were more likely to have discussed Zika virus with their provider. Because of differences perceived or real risks of Zika virus disease among different racial and ethnic groups, it is important to further investigate the communication preferences of Hispanic women. Furthermore, no information was collected on whether discussions with providers were initiated by the patient or the provider, although an attempt was made to control for differences in provider interest and awareness in Zika virus by controlling for practice location during the analysis. No significant differences were found when the unadjusted results were compared to the results adjusted for practice. However, it would be valuable in future investigations to differentiate between patient-initiated and provider-initiated discussion of topics like Zika virus.

Additionally, all practices included in study were located in the greater Atlanta area (where the CDC is located), therefore women in the study may have been more aware of the CDC and the role that the CDC plays in public health emergencies than women in other parts of the United States. Because of this and the fact that adoption of preventative measures reportedly differs by region, it would be valuable to investigate whether communication preferences for Zika virus may also differ by locale [37, 38]. However, previous studies have not found significant differences in the information-seeking preferences of pregnant women by region or country, leading the authors to believe that the results of this study are applicable beyond the metro-Atlanta area [20, 31].

## Conclusion

To our knowledge, this is the first study to explicitly examine pregnant women’s preferences for receiving communications from their prenatal care providers at the outset of a public health emergency that disproportionately affects the unborn children of pregnant women. If providers take advantage of alternative communication avenues that align with women’s communication preferences and their existing health-seeking behaviors there is an opportunity for more comprehensive and impactful communication between pregnant women and their providers.

## Additional file

Additional file 1: Public health communication preferences of pregnant women survey. (PDF 127 kb)
