# Supplementary material for: A survey-based study of Zika virus communication preferences among pregnant women in Georgia, United States
Source: BMC Pregnancy Childbirth. 2017 Sep 26;17:325. doi: 10.1186/s12884-017-1516-0 (PMC5615434; doi:10.1186/s12884-017-1516-0)
Supplement: Additional file 1: — Public health communication preferences of pregnant women survey. (PDF 127 kb) [file 12884_2017_1516_MOESM1_ESM.pdf]

Public Health Communication Preferences of Pregnant Women Survey

*****Return this survey to the nurse/receptionist when complete*****

Today's Date (MM/DD/YYYY): ________________________

Section 1. Healthcare experience

1) Is your main prenatal provider an OB/GYN or a midwife?
a) ______OB/GYN
b) ______Midwife
c) ______Don't know

2) Is your main prenatal provider:
a) _____Male
b) _____Female

3) Do you consider your prenatal provider to be your primary care doctor?
a) _____Yes
b) _____No

4) Is this your first pregnancy?
a) _____Yes
b) _____No

5) What trimester of pregnancy are you in now?
a) ______First (0 – 12 weeks)
b) ______Second (13 – 27 weeks)
c) ______Third (27 – 40+ weeks)
d) ______Don't know

Section 2. Zika and pregnancy

6) Have you heard of Zika virus?
a) ______Yes b) ______No

7) Has your prenatal care provider talked with you about Zika virus yet?
a) ______Yes b) ______No c) ______Don't know

8) How interested are you in information about Zika infection and pregnancy?

Not interested at all	A little interested	Neutral	Interested	Very interested
☐	☐	☐	☐	☐

9) Other than talking with your prenatal provider, where would you most likely go to find more information about Zika and pregnancy? Check all that apply.
a) ______Social Media (Facebook, Twitter, etc.)
b) ______My OB/GYN's practice website
c) ______CDC Website
d) ______Health department website
e) ______My friends and family
f) ______Other pregnancy-related websites (e.g. BabyCenter, What To Expect, etc.)
g) ______Other (specify)____________________

10) If your prenatal provider wanted to give you more information about Zika during pregnancy, how would you like to get that information? Check all that apply.
a) ______A "Zika virus" section on their practice website
b) ______Useful links posted on their practice Facebook page
c) ______Useful links distributed on their Twitter feed
d) ______Text messages to me
e) ______Emails to me
f) ______Educational brochures
g) ______Other (specify)_____________________

Section 3. Vaccinations during pregnancy

11) Has your prenatal provider talked with you about vaccinations during pregnancy yet?
a) ______Yes b)______No c)______Don't know

12) How interested are you in information about vaccines during pregnancy?

Not interested at all	A little interested	Neutral	Interested	Very interested
☐	☐	☐	☐	☐

13) Other than talking with your prenatal provider, where would you most likely go to find more information about vaccines during pregnancy? Check all that apply.
a) ______Social Media (Facebook, Twitter, etc.)
b) ______My OB/GYN's practice website
c) ______CDC Website
d) ______Health department website
e) ______My friends and family
f) ______Other pregnancy-related websites (e.g. www.BabyCenter.com, www.whattoexpect.com etc.)
g) ______Other (specify)_____________

14) If your prenatal provider wanted to give you more information about vaccines during pregnancy, how would you like to get that information? Check all that apply.
a) ______A "vaccines" section on their practice website
b) ______Useful links posted on their practice Facebook page
c) ______Useful links distributed on their Twitter feed
d) ______Text messages to me
e) ______Emails to me
f) ______Educational brochures
g) ______Other (specify)____________

Section 4. Safe medications and pregnancy

15) Have you talked to your prenatal provider about safe medications during pregnancy?
a) ______Yes b) ______No c) ______Don't know

16) How interested are you in information about safe medications during pregnancy?

Not interested at all	A little interested	Neutral	Interested	Very interested
◻	◻	◻	◻	◻

17) Other than talking with your prenatal provider, where would you most likely go to find more information about safe medications during pregnancy? Check all that apply.

a) ______Social Media (Facebook, Twitter, etc.)
b) ______My OB/GYN's practice website
c) ______CDC Website
d) ______Health department website
e) ______My friends and family
f) ______Other pregnancy-related websites (e.g. BabyCenter, What To Expect, etc.)
g) ______Other (specify)_____________

18) If your prenatal provider wanted to give you more information about safe medications during pregnancy, how would you like to get that information? Check all that apply.

a) ______A "Safe medications" section on their practice website
b) ______Useful links posted on their practice Facebook page
c) ______Useful links distributed on their Twitter feed
d) ______Text messages to me
e) ______Emails to me
f) ______Educational brochures
g) ______Other (specify)_____________

Section 5. Practice website & social media knowledge

19) Do you have any social media accounts? Please check all that apply.

a) _____Facebook
b) _____Twitter
c) _____Instagram
d) _____Other (specify)______________

20) Do you know if your prenatal provider has a practice website?

a) _____Yes, and I visit it regularly
b) _____Yes, but I don't visit often
c) _____Yes, but I have never visited it
d) _____No, I don't know

21) Do you know whether your prenatal provider has a practice-sponsored Facebook page?

a) _____Yes, and I have followed/liked it
b) _____Yes, but I have not followed/liked it
c) _____No, I don't know

22) Do you know whether your prenatal provider has a practice-sponsored Twitter account?

a) ______Yes, and I follow it
b) ______Yes, but I do not follow it
c) ______No, I don't know

23) If your prenatal provider started a text message service to provide you with information about prenatal health topics, how frequently would you want to get those texts?

a) ______ Twice per week
b) ______ Once per week
c) ______ Twice per month
d) ______ Once per month
e) ______ Never, I don't want that service

Section 6. Qualities of content

24) When reading information about pregnancy-related topics, how important do you find the following qualities?

	Not important	Somewhat important	Very important
**That it's endorsed or approved by the CDC**	◻	◻	◻
**That it's endorsed or approved by my own prenatal care provider**	◻	◻	◻
**That it's evidence-based**	◻	◻	◻
**That it's brief**	◻	◻	◻
**That it's important to other mothers**	◻	◻	◻

Section 7. Demographic information

25) How old are you?

a) ______18 – 24 years old
b) ______25 – 29 years old
c) ______30 – 34 years old
d) ______35 – 39 years old
e) ______40+ years old

26) What is the highest level of school that you have completed?

a) ______K-8 grade
b) ______9-11 grade
c) ______High school graduate/GED
d) ______Technical/Vocational or Associates
e) ______Bachelor Degree
f) ______Master's Degree
g) ______Doctorate

27) How would you describe your ethnic background?

a) ______African American/Black
b) ______Hispano/Latino/Chicano
c) ______Caucasian/White
d) ______Asian
e) ______Other (please specify)___________________

THANK YOU FOR COMPLETING OUR SURVEY!

PLEASE RETURN YOUR SURVEY TO THE FRONT DESK
